# Early Embryogenesis of Brown Alga *Fucus vesiculosus* L. is Characterized by Significant Changes in Carbon and Energy Metabolism

**DOI:** 10.3390/molecules22091509

**Published:** 2017-09-09

**Authors:** Elena Tarakhovskaya, Valeriya Lemesheva, Tatiana Bilova, Claudia Birkemeyer

**Affiliations:** 1 Department of Plant Physiology and Biochemistry, Faculty of Biology, St.-Petersburg State University, 199034 St.-Petersburg, Russia; st035098@student.spbu.ru (V.L.); bilova.tatiana@gmail.com (T.B.); 2 Faculty of Chemistry and Mineralogy, Leipzig University, 04103 Leipzig, Germany; birkemeyer@chemie.uni-leipzig.de

**Keywords:** Fucus, metabolomics, GC-MS, embryogenesis, zygote, brown algae

## Abstract

Brown algae have an important role in marine environments. With respect to their broad distribution and importance for the environment and human use, brown algae of the order Fucales in particular became a model system for physiological and ecological studies. Thus, several fucoids have been extensively studied for their composition on the molecular level. However, research of fucoid physiology and biochemistry so far mostly focused on the adult algae, so a holistic view on the development of these organisms, including the crucial first life stages, is still missing. Therefore, we employed non-targeted metabolite profiling by gas chromatography coupled to mass spectrometry to create a non-biased picture of the early development of the fucoid alga *Fucus vesiculosus.* We found that embryogenic physiology was mainly dominated by a tight regulation of carbon and energy metabolism. The first dramatic changes of zygote metabolism started within 1 h after fertilization, while metabolism of 6–9 days old embryos appeared already close to that of an adult alga, indicated by the intensive production of secondary metabolites and accumulation of mannitol and citric acid. Given the comprehensive description and analysis we obtained in our experiments, our results exhibit an invaluable resource for the design of further experiments related to physiology of early algal development.

## 1. Introduction

Brown algae of the order Fucales inhabit the intertidal zone of rocky seashores almost throughout the world, with the bladderwrack, *Fucus vesiculosus*, as one of the most common species. Fucoids have a broad use for humans, for instance in food, cosmetic skin treatment, industry (including algal biomass production for biofuels), phytoremediation, or as metal content bioindicators [1,2,3,4,5]. Moreover, metabolic substrates of these algae are currently extensively explored in medical research, for example related to their action upon cancer cells, their pro- and anticoagulant effects and others [6]. These multiple uses highlight the importance these organisms have to humans and explain the interest scientists all over the world develop for the physiological aspects in fucoid development.

**Figure 1:**
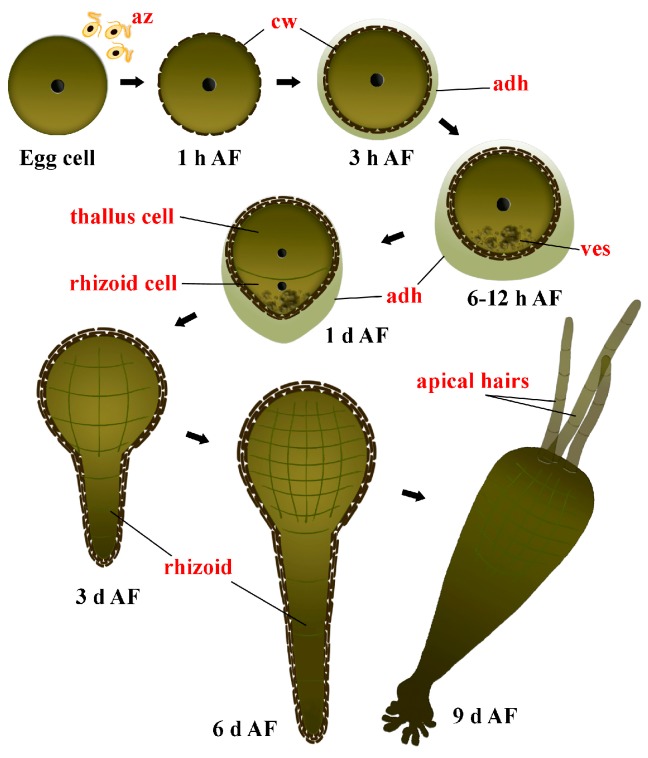
Scheme of *F. vesiculosus* early embryogenesis. **AF**—after fertilization; **az**—antherozoids; **cw**—cell wall; **adh**—adhesive material; **ves**—secretory vesicles.

Thus, fucoid embryogenesis became a generally applied model system used to study numerous aspects of algal physiology, including cell polarization, cell wall assembly, photosynthetic performance, and bioadhesion (e.g., [7,8,9,10,11]). The principal feature of this process making it so attractive to investigate is that fucoid zygotes and embryos develop independently of maternal tissues which makes them easily accessible for experimental studies at all developmental stages. The gametes of these algae are released from conceptacles into the water, where eggs are fertilized and zygotes germinate. Fucoid eggs have a spherical shape, they are initially apolar and have no cell wall (Figure 1). The first morphological sign of development can be observed within ca. 14–16 h after fertilization (AF), when a rhizoid protuberance appears on the zygote surface according to the developed polarity axis [12,13]. The polarization of fucoid zygotes has been extensively investigated (e.g., [10,14,15]). The rhizoid appearance is preceded by a series of complicated physiological processes, including asymmetric ion fluxes [10], redistribution of cytoskeletal elements [16], and targeted vesicular secretion [15]. The next critical morphological event is the first division of the zygote, which is asymmetrical and proceeds in the plane perpendicular to the formed polarity axis. Daughter cells differ distinctly in structure and fate: one of them gives rise to the thallus of the alga and the other to rhizoid [12,17]. The rhizoid cell elongates rapidly by tip growth, dividing transversely to the growth axis and creating a file of cells. During next three days (2–4 days AF), the rhizoid remains the only site of conspicuous growth in the embryo. The thallus cell undergoes a series of proliferative divisions, each division being transverse to the previous one, that gives rise to an elongated multicellular structure—the thallus part of the embryo [13,17]. In 7–8 days AF, several inner cells of the thallus part start producing specific filamentous structures—the apical hairs. Apical hair initiation is a crucial stage in fucoid embryogenesis, because subsequently the basal cell of one of these hairs becomes the apical meristematic cell of the future plant, giving rise to the apical meristem of adult alga [18,19]. Simultaneously or soon after this first sign of starting organogenesis, the primary rhizoid generates branches forming a stipe-like structure which allow the embryo to attach better to the substratum. From this moment, the basic body plan of future alga is established, so that early embryogenesis is finished after 9 days after fertilization.

Fucoids are oogamic plants, and their eggs have a reserve of storage compounds such as mannitol and laminaran. Noteworthy, fucoid eggs already can carry out photosynthesis, the efficiency of which grows rapidly during the first days after fertilization [20]. The schedule of fucoid embryogenesis is very tight—cell wall formation starts minutes after fertilization, between 3 and 6 h AF zygotes attach themselves to the substrate and become sensitive to the polarizing cues, until 12 h AF the polarity axis should be fixed etc. [9,15]. Besides the basic processes maintaining growth and development, the establishment of the elaborate mechanisms of the cell responses to the environmental factors also takes place during early embryogenesis. Environmental key factors for juvenile fucoids would be desiccation, light availability, hydrodynamic burden, grazing etc. [21,22]. Apparently, this series of complicated physiological processes going one by one or in parallel is expected to be accompanied with dramatic changes of cells’ metabolism, which, beyond ion fluxes [23,24], surprisingly, are completely unstudied despite the detailed biological studies available for this model.

Here, we make an attempt to draft the biochemical network underneath the known sequence of physiological processes using a GC-MS-based metabolomics approach. This method allows simultaneous analysis of many compounds (primary and, partially, secondary metabolites), which makes it appropriate for non-targeted characterisation of the complicated processes. Up to now, metabolomics of marine macrophytes is not among well studied fields of plant physiology. The most detailed studies of brown algae were carried out on *Ectocarpus siliculosus* [25,26,27], the genome of which was recently sequenced [28]. Several metabolite profiling studies of Laminariales and Fucales describe preferentially the dominating metabolites and compounds having potential applied relevance, such as fatty acids, pigments, and sterols [29,30,31,32,33,34,35]. These investigations are typically focused on adult algae. In contrast, the objective of our study is to compare the metabolite profiles of eggs, zygotes, and embryos of *Fucus vesiculosus* at different developmental stages. To our knowledge, this study is the first attempt to analyze detailed changes in the metabolite levels during macroalgal embryogenesis.

## 2. Results

### 2.1. General Description of Metabolic Profiles

GC-MS-based profiling of *F. vesiculosus* metabolites revealed 94 compounds identified by retention indices and spectra similarity (excluding unknowns and contaminants), represented by 38 carbohydrates (sugars, sugar-phosphates, and polyols), 13 amino acids including ten proteinogenic (Ala, Gly, Val, Ile, Pro, Ser, Thr, Glu, Asn, Phe) as well as three non-proteinogenic ones (pipecolic acid, β-alanine, 2-aminobutyric acid), 13 fatty acids and their methyl esters, six di- and tricarboxylic acids (succinic, fumaric, malic, glyceric, cis-aconitic, and citric acids), eight phenolic compounds (pyrogallol, phloroglucinol, tetrahydroxybenzol, p-hydroxybenzoic acid, homogentisic acid, difucol and diphlorethol, and a putative phloroglucinic acid/phloroglucinol derivative), and a miscellaneous group of metabolites including fucosterol, tocopherols, ascorbic acid, squalene etc. (Table S1).

**Figure 2:**
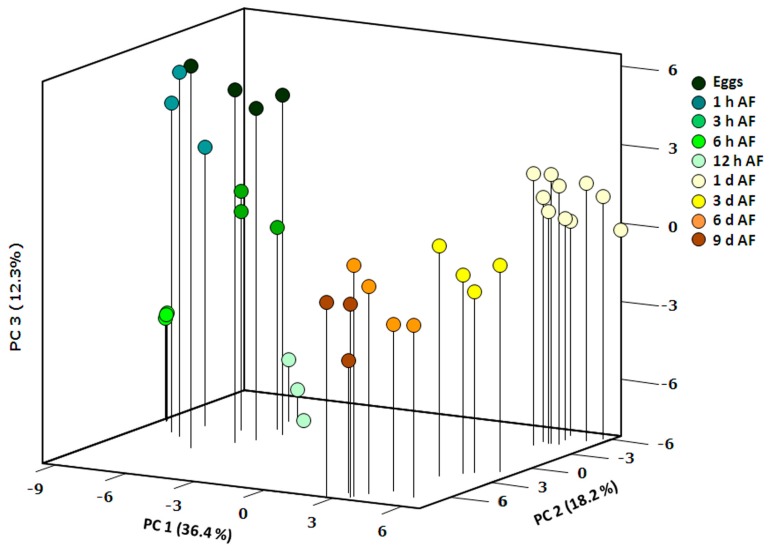
Sample scores for the first three principal components derived from PCA of the metabolite concentrations in *F. vesiculosus* eggs, zygotes, and embryos. Each developmental stage is represented with 3–10 samples. PCA was carried out with MetaboAnalyst 3.0 (http://www.metaboanalyst.ca).

The main tendencies in primary metabolite distribution and dynamics during *F. vesiculosus* embryogenesis were revealed by a principal components analysis (PCA), where the first three principal components (PCs) explain ~67% of the variance (Figure 2). A distinct clustering of cells/embryos (independent replicates) from each time point confirms the similarity of their metabolic profiles on the same developmental stage with respect to sample replicate variance in contrast to the developmental level the sample was drawn from. The first principal component describes the general trend of metabolome dynamics throughout the whole embryogenesis, splitting all the developmental stages into two distinct groups—before and after the first zygote division (ca. 20 h AF). PC 2 better explains metabolite changes in the embryos of 1–9 days AF. The physiological events specific for the first steps of the embryogenesis (0–12 h AF) are mainly reflected in the score values for PC 3. A closer examination of the PCA results suggests that separation of the 1–9 days AF and 0–12 h sample groups along PC 2 requires the second dimension of either PC 1 or PC 3, indicating that the compounds contribute to PC 2 but not to the corresponding other principal component, differentiating the two main developmental stages from each other.

The list of the highest loadings presented in Table 1 allows the identification of metabolic changes represented by the first three principal components. The compounds contributing significantly to PC 1 are mostly different sugars and polyols as well as the components of lipid metabolism (squalene, fatty acids) and, to a lesser extent, organic acids (fumarate, succinate). The most significant constituents of PC 2 are sugars, amino acids, and phenolic compounds (4-hydroxybenzoic and homogentisic acids). The phloroglucinic acid derivative, phosphorylated sugars, amino acids, and fatty acids show high loadings for PC 3. Notably, one of the major *Fucus* primary metabolites, citric acid, is in the top 10 loadings for both PC 2 and PC 3, which means it might be one of the key players throughout the whole embryogenesis studied.

### 2.2. Changes in Metabolite Profiles during Fertilization and Zygote Development

**Figure 3:**
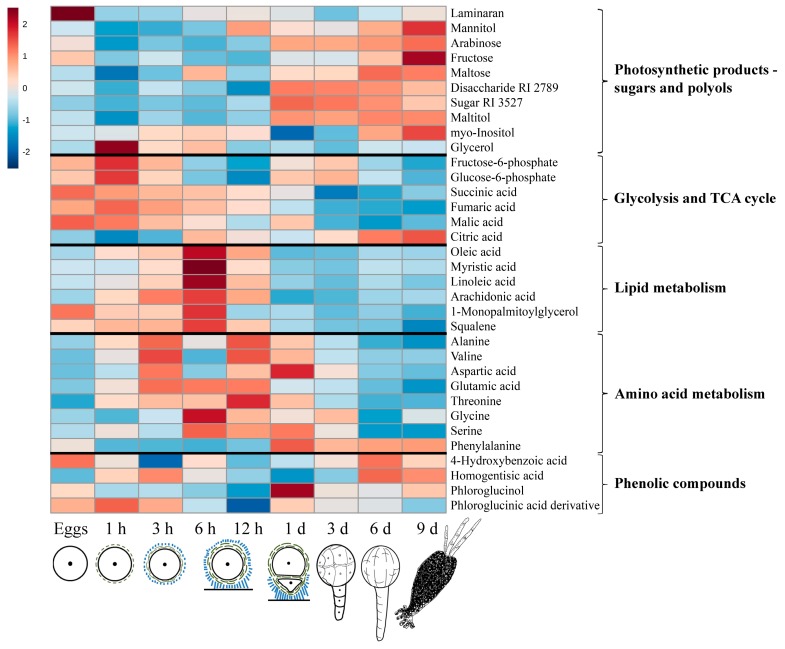
A heatmap of significantly changing key metabolites detected in *F. vesiculosus* eggs, zygotes (1 h–12 h), and embryos (1 day–9 days). Mean values of 3–10 samples are presented on a log_2_ scale. Key physiological processes of the time points: 1 h—initial cell wall deposition; 3 h—primary adhesive synthesis; 6 h—attachment to the substratum; 12 h—induction of zygote germination; 1 day—improvement of photosynthetic efficiency; 3 days—intensive rhizoid elongation via tip growth; 6 d—conspicuous growth of the thallus part; 9 days—start of organogenesis.

**Figure 4:**
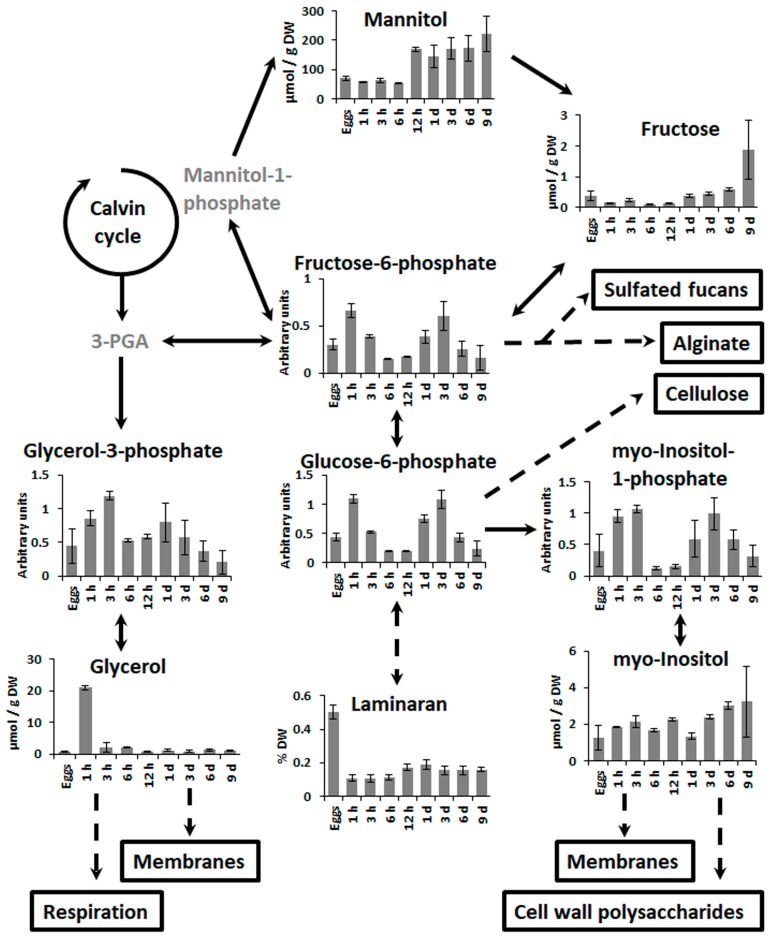
Scheme of the central carbon metabolism in brown algae. Direct reactions are presented as straight lines and reactions involving several steps are presented as dashed lines. Metabolites which were not determined are labeled in grey. Bars represent the means ± SD (standard deviation). Arbitrary units are normalized peak areas.

**Figure 5:**
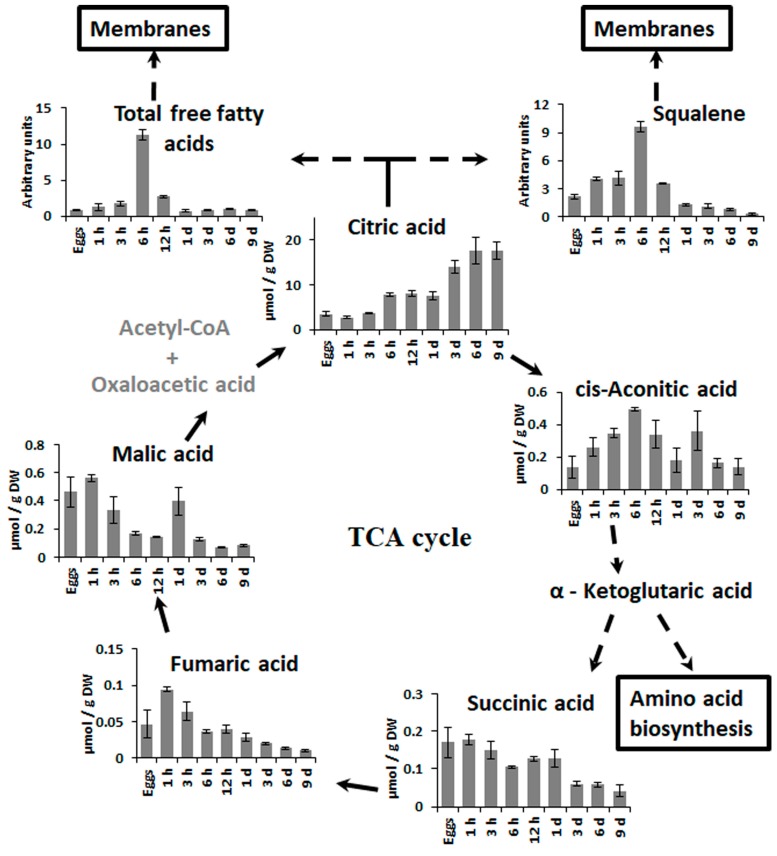
TCA cycle and presumable citric acid consumption pathways. Direct reactions are presented as straight lines and reactions involving several steps are presented as dashed lines. Metabolites which were not determined are labeled in grey. Bars represent the means ± SD. Arbitrary units are normalized peak areas.

The levels of many metabolites changed significantly during the very first hour AF (Figure 3, Figure 4 and Figure 5). Sugars and polyols generally tended to decrease, the amount of the storage compound laminaran in particular dropped down by a factor of five, and there was also a decrease in the concentrations of mannitol, maltitol, fructose, maltose etc. The only noticeable exception here was glycerol, the content of which increased dramatically, thus coinciding with the increase of the compounds associated with glycolysis and tricarboxylic acid (TCA) cycle (sugar-phosphates, fumarate, malate), except for citric acid (Figure 3). The amino acids and lipid metabolism compounds (fatty acids, squalene) also tended to increase during the first hour of zygote development.

After initial decrease, the total amount of free sugars and polyols as well as laminaran remained at approximately the same level until the first zygote division (~20 h AF). Starting from 3 h AF, sugar-phosphates and TCA-cycle dicarboxylic acids gradually decreased to levels even lower than those in the egg cells. Glycerol concentration after the prominent spike at 1 h also dropped abruptly and kept decreasing gradually throughout zygote development. The raise of the amino acid content continued, starting from 1 h AF during the first day of embryogenesis. For all of them except for glycine and serine there was a transient decrease at 6 h AF (Figure 3). All compounds associated with lipid metabolism, on the other hand, had a prominent peak at 6 h AF, after which they gradually decreased to approximately the initial level. Finally, among the phenolic compounds, phloroglucinol and the phloroglucinic acid derivative tended to decrease during the first 12 h of zygote development.

### 2.3. Changes in Metabolite Profiles in 1–9 Days Old Embryos

The period of the first zygote division is accompanied by considerable changes in metabolite profiles which are reflected in the results of the PCA (Figure 2). The compounds mainly contributing to these changes are different sugars, polyols, and components of lipid metabolism (Figure 3, Table 1). Starting from 12–24 h AF, the amount of sugars and polyols including laminaran generally increased and then mostly stopped at relatively high levels up to 9 days AF. Only mannitol, myo-inositol, and fructose augmented constantly during all the studied period of *Fucus* embryogenesis (Figure 3 and Figure 4). After the peak at 6 h AF, all the compounds associated with lipid metabolism (fatty acids, squalene) gradually decreased to the minimum level at 9 days AF (Figure 3 and Figure 5).

During 1–9 days of embryogenesis, compounds associated with respiration generally exhibited a lower abundance with a spike of sugar-phosphates at the third day. The only exception was citric acid, the concentration of which increased constantly during all the studied embryogenic stages. Amino acids mostly reached their maximum at 12–24 h AF, and then returned to approximately their initial level in the egg cells (Figure 3). Compared to the zygotes, *Fucus* embryos contained more phenolic compounds. The most noticeable event here was a spike of phloroglucinol at 1 day AF (Figure 3).

## 3. Discussion

### 3.1. General Considerations

This study presents the first characterization of the dynamic metabolic profile during fucoid embryogenesis. The list of metabolites identified in *Fucus* embryos (Table S1) is generally in good accordance with the results of previous biochemical reports on brown algae [26,29,33,35,36,37,38], but also contains compounds reported for the first time in fucoids. The most abundant soluble carbohydrate is mannitol, whereas the other free sugars and polyols (glucose, fructose, sucrose, myo-inositol) are present in significantly lower quantities. Most probably this is a result of their rapid conversion into storage compounds such as mannitol and laminaran via hexose-phosphates (Figure 4). The predominant organic acids are citrate and malate (Figure 5, Table S1). A detailed study made on Ectocarpus [26] also showed high level of citrate, but undetectable quantity of malate. We suppose that it might be the same for adult *F. vesiculosus* thalli, where the final stages of early embryogenesis are accompanied by citrate accumulation and malate decrease (Figure 3 and Figure 5). In plant cells, malate participates in anaplerotic reactions in mitochondria replenishing the citrate and α-ketoglutarate loss from TCA cycle for amino acid and fatty acid biosynthesis. Malate is oxidized to pyruvate, generating NADH via NAD-malic enzyme in the mitochondrial matrix thus allowing citrate to be formed without operating the complete TCA cycle. Probably, a stored pool of malate in fucoid eggs contributes to active biosynthetic processes in the first days of embryogenesis. Among the free proteinogenic amino acids detected in *F. vesiculosus* eggs, zygotes, and embryos, the most abundant are alanine, glutamate, aspartate, and serine (Table S1). Generally, amino acid profiles are in accordance with literature data for different brown algae and resemble the profiles observed in higher plants [26,39].

Besides the common, primary plant cell metabolites, *F. vesiculosus* profiles contain a large amount of sugars and polyols with relatively high molecular weight, which are usually difficult to identify unambiguously (Table S1). Most probably, the majority of them represents different products of hydrolysis of the long-term carbon storage compound laminaran, which is a polydisperse polymer based on β-1,3-glucose and containing up to 3.7% mannitol [40,41].

### 3.2. Zygote Development

Many key physiological processes take place during the single-cell stage of fucoid embryogenesis (first ~20 h AF). The specificity of this developmental stage is reflected in the PCA results of our metabolite profiling data, where the first principal component distinctly merges all the scores related to eggs/zygotes (till 12 h AF) separating them from the embryos (Figure 2). The most significant physiological events at this stage are: egg fertilization, initial cell wall synthesis in the zygote, attachment to the substratum, cell polarization according to environmental cues, and formation of the rhizoid protuberance (zygote germination) [14,15]. Our data implies that biochemical processes underlying these events lead to changes in the content of compounds associated with respiration and lipid and amino acid metabolism (Figure 2 and Figure 3, Table 1).

Unfertilized fucoid eggs can survive in seawater for several days, and at least 50% of them can be successfully fertilized within 48 h after releasing out of oogonia (data not shown). They can already photosynthesize, though the intensity of photosynthesis is much lesser than in several days old embryos [7,20,42]. These limited photosynthetic capacities and potential ability of fucoid embryos to develop to some extent in the darkness imply that storage compounds concentrated in the egg cells should be the main source of carbon and energy during early embryogenesis [43,44]. The two most important carbon storage compounds in brown algae are the sugar alcohol mannitol, which is also a principal, stable product of photosynthesis, and β-1,3-glucose polymer laminaran [25,40]. Though the detailed mechanism of remobilization of laminaran in algae is still debated, the data of metabolite profiling confirm that the key event of the first hour AF is the diversion of carbon fluxes from laminaran, mannitol, and other polyols and sugars (fructose, arabinose, disaccharides) into more metabolically active substances—sugar-phosphates and glycerol (Figure 3 and Figure 4), which is the widely accepted notion for laminaran remobilization. Consequently, laminaran content decreases 5-fold for the first hour of zygote development (Figure 4)—this result is in agreement with the data of Quatrano and Stevens (1976), who reported a laminaran decrease in 7 h AF [43]. Relative change in mannitol concentration is not as big (though statistically significant), most probably because this substance is also used as compatible solute and should not change dramatically to maintain constant osmotic conditions [45]. However, Allen et al. (1972) showed a considerable increase of internal osmotic pressure in just fertilized fucoid zygotes, which might be a result of K^+^ and Cl^−^ influx [23]. During the first three hours after fertilization, cell wall is formed, and thus the eggs are necessarily at or near osmotic equilibrium with the sea water. After that, the internal osmotic pressure increases rapidly and reaches its equilibrium by the start of germination. Our data further suggests that glycerol accumulation may contribute to maintaining early osmotic equilibrium with the sea water before K^+^ and Cl^−^ influx starts, particularly during the initial phase of cell wall formation.

The general decrease of sugar and polyol content coincides with accumulation of substances associated with respiration (Figure 3), and it is known that respiration intensity increases significantly during the first hours of fucoid embryogenesis [46,47]. However, metabolite profiling data also shows a more pronounced change in the content of sugar-phosphates (fructose-6-phosphate, glucose-6-phosphate, myo-inositol-1-phosphate) than in the content of TCA cycle intermediates, which are mostly quite stable or slightly decreasing during the first hour AF, and only fumaric acid demonstrates a significant increase (Figure 3 and Figure 5). This may be a result of an increased respiration rate when there is no accumulation of TCA cycle metabolites because of their high demand. Also, this may mean that a considerable part of sugar-phosphates is consumed not in respiration, but in biosynthetic reactions. It was shown before that these compounds are at the crossroads of different metabolic pathways, being the key elements of central carbon metabolism in brown algae [25]. In tissues of adult *F. vesiculosus* thalli, sugar-phosphates, glycerol, and glyceric acid are the main metabolites contributing to rapid carbon distribution [48]. A pathway leading to formation of alginate and sulfated fucans starts from fructose-6-phosphate [49,50], while glucose-6-phosphate may be consumed for cellulose biosynthesis [51] and myo-inositol-1-phosphate is an initial precursor of different cell wall polysaccharides [52]. All together, these biosynthetic processes provide materials for cell wall formation and wall deposition on the surface of fucoid zygotes starting within several minutes after fertilization [8]. It was shown that alginates are the first wall constituents appearing on the cell surface, and cellulose can be detected within ~20 min AF and sulfated fucans within 1 h AF [43,53]. Another process which evidently starts in parallel with cell wall deposition is a secretion of adhesive material, the composition of which is similar to wall matrix [11]. Attachment to the substratum is one of the most urgent tasks for developing zygotes, a prerequisite for perception of polarizing environmental stimuli [9,11]. Both processes, intensive cell wall deposition and adhesive formation, last until ~6 h AF, when the composition of the cell wall becomes stable and the irreversible rigidification of the adhesive substance takes place [11,43,53]. Besides precursors of cell wall and adhesive components, this enhanced vesicular secretion requires a pool of membrane constituents. Metabolite profiling data show a constant increase of the compounds associated with lipid metabolism during the first 6 h of zygotes development (Figure 3 and Figure 5). The observed discrepancy in the dynamics of TCA cycle components—the reverse behavior of citric acid and dicarboxylic acids (Figure 3)—may also be the result of intensive lipid turnover in the first hours of *Fucus* embryogenesis. Citric acid is one of the central metabolites in plant biochemistry mediating the crosstalk of different pathways such as the TCA cycle, the glyoxylate cycle, and lipid biosynthesis. Thus, the decrease of citric acid content in 1 h AF persisting during next 2 h within the context of relatively enhanced concentration levels of the other TCA metabolites might be attributed to its consumption for fatty acids and squalene biosynthesis, and then membrane formation (Figure 5).

As soon as fucoid zygotes attached themselves to substratum they become sensitive to a series of environmental cues, according to which they form their initial polarity axis and start their differentiation process [12]. As zygote differentiation (first of all—tip growth initiation, which occurs ca. 12 h AF) also needs active vesicular transport based on membrane constituents [8,54], we suppose that after 6 h of development, when cell wall formation is mostly ready, it would be the main sink for fatty acids and squalene. So, the dramatic increase of compounds associated with lipid metabolism in the period around 6 h AF (Figure 3 and Figure 5) apparently supports all three processes—cell wall deposition, adhesive formation, and zygote differentiation.

Quatrano (1978) showed that the proteins required for first zygote division and rhizoid formation are synthesized approx. between 8 and 13 h AF [12]. De novo mRNA synthesis starts in fucoid zygotes within an hour AF, being a prerequisite for subsequent polarization and growth processes [12,14]. This coincides with the increase of amino acid content during the first hours of zygote development (Figure 3). The high demand of the TCA metabolite α-ketoglutarate for amino acid biosynthesis may also contribute to low citrate level at this stage. We could barely detect α-ketoglutarate in the embryo metabolite profiles—most probably because of its rapid consumption. The temporary drop in concentration of most amino acids in the period between 3 and 6 h AF might be the result of their massive consumption for protein biosynthesis supporting the primary events of this embryogenic stage. Only two amino acids, namely glycine and serine, increase significantly during this period. The general feature of these two amino acids is that they both are involved in photorespiration. Presumably, the appearance of the full-fledged cell wall makes the zygote rearrange its metabolism because the wall hampers gas exchange and glycolate diffusion, thus leading to activation of photorespiration reactions. So, the period around 6 h AF might be the time of the cell’s adaptation to the changed diffusion rates. At 12 h AF, most of the amino acids again are upregulated, apparently to support the new wave of the intensification of protein synthesis.

### 3.3. Embryogenesis after the First Zygote Division

According to the anatomical and physiological data, the first days of fucoid embryogenesis after zygote division include such principal processes as improvement of photosynthetic efficiency, intensive rhizoid elongation, and start of organogenesis [17,19,20]. Metabolite profiling data shows that the most noticeable difference between zygotes and embryos is the content of photosynthetic products—sugars and polyols—from 1 day AF on (Figure 3 and Figure 4). Mannitol concentration significantly increases as well as different mono-, di-, and trisaccharides. It is known that the photosynthetic activity of fucoid embryos grows rapidly during the first days of embryogenesis [7,20,42]. Maximum electron transport rate in *F. vesiculosus* embryos increases more than twice for the first three days of development [20]. We may conclude that sugar and polyol accumulation starting soon after the first zygote division means that photosynthesis became effective enough to fill up energy and material expenses and initiate resource storage for subsequent development.

The content of the compounds associated with respiration, except citrate, generally goes down, presumably reflecting the improvement of photosynthesis efficiency (Figure 3 and Figure 5). A spike of sugar-phosphates at day 3 coincides well with the period of fast rhizoid elongation via apical cell tip growth. Tip growth is a highly energy- and substrate-dependent process based on the constant production and redistribution of numerous membrane and cell wall components [55]. As there is no synchronous increase of the other respiration-associated compounds, this sugar-phosphate spike is most probably connected with intensification of biosynthetic processes supporting rhizoid elongation.

According to PCA loadings, phenolic compounds represent one more group of metabolites contributing significantly to the difference between the *Fucus* embryogenesis stages (Table 1). There is a general drop of the phenolic compounds during 6–12 h followed by accumulation in 3–6 days old embryos with a spike of phloroglucinol in 1-day old embryos (Figure 3). As secondary metabolism in brown algae is still poorly investigated, this part of the results is most interesting, but difficult to interpret. Phloroglucinol is a monomer of a specific group of brown algal phenolic compounds—phlorotannins [56]. The presumable spectrum of physiological functions of these polyphenols includes antioxidative activity and chemical defense against biofouling and grazing [22,57]. Also, they are integral components of fucoid cell walls and adhesive material [53,58]. Although the cell wall composition is stabilized until 6 h AF, its thickness is still growing until 12 h AF [43], which means persisting consumption of wall constituents. Polyphenolics are deposited preferentially into the wall of rhizoid protuberance of the germinating zygote, thus being among the last components incorporated into the zygotic cell wall [59]. So, the gradual decrease of free phloroglucinol during 1–12 h AF (Figure 3) could be a result of its rapid polymerization and incorporation into cell wall. Temporary phenolics accumulation after 12 h AF causing a spike at 1 day AF might be a sign of finalizing cell wall formation. The synchronous changes in phloroglucinol and the phloroglucinic acid derivative content, the latter with its lowest preceding the highest phloroglucinol concentration, followed by upregulation of the other two detected phenolic compounds during this period of embryogenesis imply that these compounds should be metabolically linked. However, up to now, phlorotannin biosynthetic pathways have been little characterized at the biochemical and molecular levels [60], and a possible role of phloroglucinic acid, whether as a byproduct, precursor, or product of phloroglucinol needs further investigation. Phloroglucinic acid and its methyl ester have been earlier isolated from several higher plants [61], but to our knowledge it is the first hint on the potential presence of this compound in brown algae.

Generally, after 3 days of development, the metabolic profiles of the embryos look more and more like those of the adult brown algae. The content of amino acids, fatty acids, and squalene decreases significantly (Figure 3 and Figure 5). Among the organic acids of the TCA cycle, there is a considerable accumulation of citric acid, which is particularly noticeable in 6–9 days old embryos. This organic acid tends to dominate the metabolic profiles of adult brown algae [26], including fucoids [38]. Presumably, its low level in the zygotes and young embryos is a specific biochemical feature of this developmental stage, when citric acid is massively consumed for biosynthetic reactions, such as membrane formation and amino acid biosynthesis.

## 4. Materials and Methods

### 4.1. Plant Material Collection and Culturing

Mature receptacles of *Fucus vesiculosus* L. were collected in the Keret Archipelago (Kandalaksha Bay, White Sea), washed with seawater, dried with filter paper, and stored in the dark at 4 °C for up to 2 weeks. Collection of gametes and fertilization were accomplished according to [14,62]. Fertilization was defined as 15 min after mixing the suspensions of eggs and antherozoids. Immediately after fertilization, the zygotes were divided into several groups and placed into 60-mm plastic Petri dishes in Millipore (pore size 0.45 μm) filtered seawater at 15 °C. Later, the developing zygotes and embryos were kept in Petri dishes with seawater under continuous light provided by cool white fluorescent bulbs at an irradiance of 20–25 μmol photons m^−2^ s^−1^. The germination rate for all the samples was more than 90%.

### 4.2. Sample Preparation

Before starting the sample preparation, the feasibility and appropriate sample amount were tested in an initial experiment with ascending amounts of algal material. Samples for final metabolite profiling were taken within 2 h after releasing out of oogonia (unfertilized eggs), at 1, 3, 6, and 12 h AF (zygotes) and then at 1, 3, 6, and 9 days AF (embryos) as independent replicates. 20 mg FW of *F. vesiculosus* eggs, zygotes, or embryos was harvested by filtering through 40 μm cell strainers (BD Biosciences), immediately poured with cold methanol (−25 °C), quickly ground in pre-cooled manual glass tissue grinder and left in 1 mL of cold methanol for extraction. The embryos and zygotes already attached to the Petri dishes were gently detached with the plastic cell scraper before filtering. Five hundred μL of methanol extracts was transferred to clean 1.5 mL polypropylene Eppendorf tubes (VWR, Dresden, Germany) and vacuum-dried for subsequent chromatographic analysis.

### 4.3. Metabolite Profiling

Metabolite profiling analyses were carried out according to Hutschenreuther et al. (2012) [63]. Briefly, vacuum-dried extracts were incubated by shaking in methoxyamine hydrochloride (Sigma-Aldrich Chemie GmbH, Taufkirchen, Germany) in pyridine and *N*,*O*-Bis(trimethylsilyl)-trifluoroacetamide (Macherey-Nagel GmbH and Co KG, Düren, Germany). After derivatization, samples were transferred to glass vial micro-inserts and subjected to GC-MS analysis on a Trace GC Ultra equipped with an A200S autosampler (CTC Analytics, Zwingen, Switzerland) and coupled with a MAT95 XP double focusing sector field mass spectrometer (MS), (Thermo Electron, Bremen, Germany) with standard electron impact ionization. Within each sequence, a mixture of alkanes (C_10_–C_32_) in hexane was measured for the calculation of Kovats retention indices (RI) [64]. A mix of authentic standards containing 20 amino acids, 20 sugars and polyols, 19 organic acids, and phloroglucinol, was co-spiked to confirm the identity of expected compounds.

Peak deconvolution was accomplished using AMDIS 2.65 [65]. The retention indices (RIs) were automatically calculated using an AMDIS calibration file containing the batch retention times of each alkane. GMD (Golm metabolome database, GMD_20100614_VAR5_ALK, 24.09.2010, [66]) and NIST14 (National Institute of Standards and Technology, Gaithersburg, MD, USA) were used for identification of the peaks based on spectra comparison. Quantitation of metabolites was performed by peak integration of the corresponding extracted ion chromatograms (*m*/*z* ± 0.5) for representative intense signals at specific retention times (RT) using Xcalibur 2.0.7. Where applicable, calibration was performed by the standard addition method using five calibration levels.

Laminaran was extracted from 5–8 mg dried homogenized algal material, and quantified based on the enzymatic assay described by Gómez and Wiencke (1998) [67].

### 4.4. Statistical Analysis

The measurements were performed with three to ten replicates. Excel 2013 (Microsoft, Redmond, Washington, DC, USA) and MetaboAnalyst 3.0 Web application (http://www.metaboanalyst.ca) were used for data processing and normalization procedures, principal component analysis (PCA), creation of figures, and metabolic heatmap construction [68]. The data processing included peak area normalization to the median of all areas within a chromatogram, generalized logarithm transformation, and mean centered data scaling. All values are expressed as means and standard deviations.

## 5. Conclusions

The present study, for the first time, provides results from analysis of detailed changes in metabolite concentration during macroalgal embryogenesis. We conclude from this newly available data that, generally, the appearance of the metabolic profiles is in good accordance with the common knowledge about the anatomy and physiology of fucoid embryogenesis. The results of principal component analysis imply that from a biochemical perspective, *Fucus* early embryogenesis can be divided into two stages: (i) the *zygote development*, with the first crucial metabolic changes compared to the eggs, starting at least as early as 1 h after fertilization and characterized by high concentrations of monosaccharides and common organic and fatty acids; and (ii) the *embryo development*, starting after the first zygote division (after 24 h AF) and associated with the accumulation of storage compounds, presumably as a consequence of improved photosynthetic efficiency. Finally, at the end of the second stage (9 days AF), the embryos already metabolically resemble the adult plants.

A major advantage of a metabolomics approach is the identification of compounds serving as biochemical markers, here as markers of different embryonic stages. As such, the egg cells are characterized by a generally low metabolic diversity, only the substrates of glycolysis and the TCA cycle were found to be abundant, potentially enabling an immediate supply of energy and biosynthetic substrates for the following dramatic metabolic changes. Thus, the first hour of *zygote development* can then be understood as a transition state to further activate these fundamental pathways and deliver the subsequently required first basic biosynthetic substrates while balancing the osmotic potential of the cell. These processes are linked to an increase of sugar-phosphates and glycerol, which is followed by upregulation of amino acids and compounds associated with lipid metabolism. It can be expected that the processes of fucoid embryogenesis are most vulnerable to environmental or anthropogenic influences during this stage (within the first day after fertilization). Therefore, *zygote development* might be a good starting point for research related to the reasons for the decline or prosperity of Fucales, both important for environmental protection of the algae or its breeding for commercial uses. The ensuing stage of *embryo development* starts with a spike of phloroglucinol 1 day AF and subsequent upregulation of phenolic compounds, while accumulation of mannitol, myo-inositol, fructose, and citric acid marks the end of early embryogenesis (9 days AF); this stage seems to be mostly related to the finalization of the cell wall and incipient production of carbohydrate polymers and secondary metabolites. In summary, embryogenesis is mainly characterized by a temporary redirection of the carbon flow from respiration to biosynthesis processes with the sugar-phosphates and citric acid as crucial key metabolites differentially regulated at almost every step of zygote and embryo development.

Our data also reveals the potential importance of substrates whose metabolic role is not fully elucidated yet for fucoid embryogenesis. In particular, the metabolite profiles of the embryogenic stages highlight the importance of the biosynthesis of phloroglucinol-derived substances and the role of other, so far undetected in *Fucus vesiculosus* phenolic compounds, such as homogentisic acid and *p*-hydroxybenzoic acid. Here, a detailed target analysis of metabolic changes before and directly after 1 day AF could uncover valuable hints for the elucidation of the biosynthesis of these crucial secondary metabolites of brown algae, an ongoing research aim in our lab.

As a general conclusion, our data exhibits an essential resource for the research community related with algal physiology.

## Figures and Tables

**Table 1:** The compounds with the highest loadings (absolute values) in the first three principal components. The vertical order in the table is according to the absolute values of the loadings—for each PC the compound with the highest value is in the first line. Signs in the brackets indicate positive or negative values.

**PC 1**	**PC 2**	**PC 3**	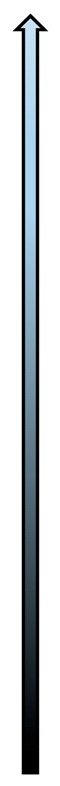
Squalene (−)	Trisaccharide RI 3415 (−)	Phloroglucinic acid derivative (+)	
Sugar RI 3527 (+)	Serine (−)	Glucose-6-phosphate (+)	
Polyol RI 3514 (+)	Disaccharide RI 2669 (−)	1-Octadecanol (+)	
Maltitol (+)	Glucose (−)	Glycine (−)	
Disaccharide RI 2789 (+)	Myo-Inositol (+)	Dodecanoic acid methylester (+)	
Fumaric acid (−)	Fructose (+)	Myo-Inositol-1-phosphate (+)	
Galactose (+)	Citric acid (+)	Eicosanoic acid (+)	
Xylose (−)	Trisaccharide RI 3447 (−)	Threonine (−)	
Arabinose (+)	Aspartic acid (−)	Fructose-6-phosphate (+)	
Oleic acid (−)	4-Hydroxybenzoic acid (+)	Citric acid (−)	
1-Monopalmitoylglycerol (−)	Threitol (−)	Malic acid (+)	
Disaccharide RI 2851 (+)	Alanine (−)	Isoleucine (−)	
Phenylalanine (+)	Pentitol RI 1685 (+)	β-alanine (−)	
Linoleic acid (−)	Glyceric acid (+)	1-Eicosanol (+)	
Polyol RI 3482 (+)	Pipecolic acid (−)	Oleic acid (−)	
Arachidonic acid (−)	Homogentisic acid (+)	Linoleic acid (−)	
Sucrose (−)	Maltose (+)	Glutamic acid (−)	
Succinic acid (+)	Phytol (+)	Arachidonic acid (−)	
Phosphoric acid (+)	Polyol RI 3466 (−)	Pipecolic acid (−)	
Polyol RI 3458 (+)	Tocopherol γ (+)	Myristic acid (−)	

## Supplementary Materials

The following are available online, Table S1: Retention times (RT), calculated retention indexes (RI) and content of compounds detected in *F. vesiculosus* eggs, zygotes, and embryos.
