# Supplementary material for: Early Embryogenesis of Brown Alga Fucus vesiculosus L. is Characterized by Significant Changes in Carbon and Energy Metabolism
Source: Molecules. 2017 Sep 9;22(9):1509. doi: 10.3390/molecules22091509 (PMC6151410; doi:10.3390/molecules22091509)
Supplement: Supplementary file 1 [file molecules-22-01509-s001.pdf]

Table S1. Retention times (RT), calculated retention indexes (RI) and content of compounds detected in *F. vesiculosus* eggs, zygotes and embryos. All values of metabolite content represent the means of 4 replicates ± standard deviation. DW = "dry weight"

**Primary metabolites**					
**No.**	**Analyte**	**RT (min)**	**RI**	**Quantification, *m/z* ± 0.5**	**µmol/g DW in *F. vesiculosus* eggs**
1	Alanine	10.61	1106	116	38±10
2	2-Aminobutyric acid	12.18	1172	130	-
3	Valine	13.15	1214	218	2.9±1.1
4	Glycerol	14.41	1268	218	0.66± 0.07
5	Phosphoric acid	14.45	1272	314	-
6	Isoleucine	14.88	1288	158	0.8±0.5
7	Proline	15.01	1295	142	0.6±0.5
8	Glycine	15.16	1303	248	0.9±0.2
9	Succinic acid	15.41	1312	247	0.17±0.04
10	Glyceric acid	15.67	1324	292	-
11	Fumaric acid	16.19	1348	245	0.05±0.02
12	Serine	16.32	1354	218	5.8±4.0
13	Threonic acid-1,4-lactone	16.74	1373	101	-
14	Threonine	16.87	1379	218	0.6±0.3
15	β-alanine	17.78	1422	290	-
16	Malic acid	19.06	1483	233	0.50±0.10
17	Threitol	19.38	1498	217	-
18	Aspartic acid	19.69	1514	233	3.9±2.1
19	Pyroglutamic acid	19.89	1524	258	-
20	Threonic acid	20.44	1554	220	-
21	Methyldodecanoate	21.57	1612	83	-
22	Glutamic acid	21.62	1618	348	12.5±4.6

23	Phenylalanine	21.80	1626	192	3.3±1.0
24	Xylose	22.26	1649	217	
25	Arabinose	22.39	1655	217	0.030±0.004
26	Unknown pentitol	22.95	1685	319	-
27	Unknown pentitol	23.13	1694	307	-
28	Isocitric lactone	23.51	1716	303	-
29	cis-Aconitic acid	24.06	1746	229	0.14±0.06
30	Glycerol-3-phosphate	24.24	1757	358	-
31	Citric acid	25.24	1814	211	3.5±0.4
32	Myristic acid	25.81	1847	300	-
33	Fructose	26.05	1862	307	0.37±0.14
34	Galactose	26.27	1875	319	0.02±0.01
35	Mannose	26.40	1882	319	0.8±0.3
36	Glucose	26.48	1887	364	23±7
37	Mannitol	26.95	1916	421	70± 8
38	Unknown sugar	27.64	1958	333	-
39	Unknown hexose	27.88	1972	129	-
40	Gluconic acid	28.07	1985	333	-
41	Palmitic acid	29.01	2046	328	-
42	myo-Inositol	29.49	2078	432	1.3±0.7
43	Methyloctadecenoate	29.94	2108	264	-
44	1-Octadecanol	30.61	2153	111	-
45	Linoleic acid	31.47	2211	352	-
46	Oleic acid	31.55	2217	354	-
47	Fructose-6-phosphate	32.62	2294	315	-
48	Glucose-6-phosphate	32.79	2306	387	-
49	1-Eicosanol	33.36	2349	355	-

50	Arachidonic acid	33.65	2368	361	-
51	Myo-Inositol-1-phosphate	34.02	2394	387	-
52	Eicosanoic acid	34.56	2436	435	-
53	Unknown disaccharide	36.17	2564	361	-
54	1-Monopalmitoylglycerol	36.37	2579	459	-
55	Sucrose	36.74	2612	437	<0.01
56	Docosanoic acid	37.04	2634	397	-
57	Unknown disaccharide	37.44	2669	361	-
58	Maltose	37.98	2717	361	0.120±0.003
59	Unknown polyol	38.47	2758	451	-
60	Unknown disaccharide	38.84	2789	453	-
61	Unknown disaccharide	39.25	2824	481	-
62	Maltitol	39.44	2841	525	-
63	Unknown disaccharide	39.57	2851	571	-
64	Unknown sugar	39.73	2865	409	-
65	Unknown trisaccharide	46.19	3415	361	-
66	Unknown trisaccharide	46.57	3447	361	-
67	Unknown polyol	46.69	3458	361	-
68	Unknown polyol	46.79	3466	361	-
69	Unknown polyol	46.99	3483	361	-
70	Unknown polyol	47.34	3514	525	-
71	Unknown sugar	47.50	3527	345	-

Secondary metabolites

**Nr**	**Analyte**	**Ref***	**RT**	**RI**	**Quantification, *m/z* ± 0.5**	**µmol/g DW in *F. vesiculosus* eggs**
1	Benzoic acid	[1,2]	14.04	1252	179	-
2	Pipecolic acid	-	16.48	1361	156	-
3	2,2-Biphenol	-	20.72	1567	330	-

4	Pyrogallol	-	21.29	1598	342	-
5	p-Hydroxybenzoic acid	[3,4]	21.90	1629	267	-
6	Phloroglucinol	[5,6]	22.12	1641	342	0.11±0.01
7	Tetrahydroxybenzol	[7]	24.17	1752	430	-
8	Homogentisic acid	[7]	25.56	1832	341	-
9	Phytol	[2]	25.65	1838	123	-
10	Dehydroascorbic acid dimer	[8,9]	25.77	1845	406	-
11	Ascorbic acid	[8,9]	27.51	1950	299	-
12	Pentadecanoic acid	[2,10,11]	27.56	1953	332	-
13	Difucol	[12]	32.44	2281	682	-
14	Diphlorethol	[13]	33.83	2380	610	-
15	Phloroglucinat/ phloroglucinol derivative	-	34.83	2459	443	-
16	Tetracosenoic acid	-	39.00	2803	423	-
17	Squalene	[10,14]	39.23	2822	137	-
18	Tocopherol δ	[8,15]	40.11	2897	209	-
19	Phloroglucinol derivative	-	40.89	2963	476	-
20	Tocopherol β	[8,15]	41.05	2977	489	-
21	Tocopherol γ	[8,15]	41.17	2988	489	-
22	Tocopherol α	[8,15]	42.69	3117	277	-
23	Unknown sterol	-	42.91	3136	459	-
24	Fucosterol	[16]	44.92	3307	327	-
25	Putative phloroglucinol trimer[#]	-	47.28	3502	609	-
26	Putative phloroglucinol trimer[#]	-	47.57	3530	609	-

*Ref = references; references are given with respect to at least one reported occurrence in brown algae. According to this, five tentatively identified analytes have not been reported in any species of brown algae yet.
[#]e.g. fucophlorethols[17], or triphlorethol, trifuhalol

[1] Z. Kamenarska, A. Ivanova, R. Stancheva, M. Stoyneva, K. Stefanov, S. Dimitrova-Konaklieva, S. Popov, *Botanica Marina* **2006**, *49*, 343.

[2] Z. Kamenarska, S. Dimitrova-Konaklieva, K. Stefanov, H. Najdenski, I. Tzvetkova, S. Popov, *Botanica Marina* **2002**, *45*, 623.

[3] E. P. Moraes, F. J. Rupérez, M. Plaza, M. Herrero, C. Barbas, *Electrophoresis* **2011**, *32*, 2055.
[4] L. Onofrejová, J. Vasícková, B. Klejdus, P. Stratil, L. Misurcová, S. Krácmar, J. Kopecký, J. Vacek, *Journal of pharmaceutical and biomedical analysis* **2010**, *51*, 464.
[5] T. Nakamura, K. Nagayama, K. Uchida, R. Tanaka, *Fisheries Science* **1996,** *62*, 923.
[6] K.-W. Glombitza, H. U. Rosener, H. Vilter, W. Rauwald, *Planta Medica* **1973**, *24*, 301.
[7] K.-W. Glombitza, Shu-Ming Li, Phytochemistry , 30, 2741-2745,
[7] E. Rickert, M. Wahl, H. Link, H. Richter, G. Pohnert, *PloS one* **2016**, *11*, e0168196.
[8] S. Lordan, R. P. Ross, C. Stanton, *Marine drugs* **2011**, *9*, 1056.
[9] I. M. Munda, *Hydrobiologia* **1987**, *151-152*, 477.
[10] M. E. Hattab, H. S. S. A. Easa, A. Tabaries, L. Piovetti, J.-M. Kornprobst, *Journal of Essential Oil Research* **2007**, *19*, 37.
[11] S. V. Khotimchenko, *Phytochemistry* **1995,** *38, 1411*.
[12] K.-W. Glombitza, H.-W. Rauwald, G. Eckhard, Phytochemistry. 1975, 14, pp. 1403-1405.
[13] K.-W. Glombitza, H. U. Rosener, D. Müller, Phytochemistry. 1975, 14, pp. 1114-1116.
[14] P. Deepak, R. Sowmiya, G. Balasubramani, P. Perumal, *Journal of Taibah University Medical Sciences* **2017**, *12*, 329.
[15] T. Nakamura, K. Nagayama, S. Kawaguchi, *Fisheries Science* **1994,** *60*, 793.
[16] L. J. Goad, T. W. Goodwin, *European Journal of Biochemistry*, **1969**, *7*, 502.
[17] J. S. Craigie, A. G. McInnes, M. A. Ragan, J. A. Walter, *Canadian Journal of Chemistry,* **1977***,* *55*, 1575.
